# RETRACTED: Wang et al. HSP70–eIF4G Interaction Promotes Protein Synthesis and Cell Proliferation in Hepatocellular Carcinoma. *Cancers* 2020, *12*, 3410

**DOI:** 10.3390/cancers18060891

**Published:** 2026-03-10

**Authors:** Meng Wang, Kai Wei, Baifeng Qian, Svenja Feiler, Anastasia Lemekhova, Markus W. Büchler, Katrin Hoffmann

**Affiliations:** Department of General, Visceral, and Transplantation Surgery, Ruprecht Karls University, Im Neuenheimer Feld 110, 69120 Heidelberg, Germany; mengwang199310@gmail.com (M.W.); doctorweikai@163.com (K.W.); mike1232006@126.com (B.Q.); svenja.feiler@gmx.net (S.F.); anastasia.lemekhova@med.uni-heidelberg.de (A.L.);

The journal retracts the article titled, “HSP70–eIF4G Interaction Promotes Protein Synthesis and Cell Proliferation in Hepatocellular Carcinoma” [1]. This retraction supersedes the correction previously published for this article [2].

Following publication, concerns were brought to the attention of the Editorial Office regarding the integrity of a number of figures presented in this article [1].

In accordance with the journal’s standard procedures, the Editorial Office and Editorial Board conducted an investigation that identified duplications between Figure 3 and Figure 4, as well as within Figure 4. Although the authors cooperated with the investigation, the explanations and supporting materials provided were not deemed sufficient by the Editorial Board to resolve the identified concerns. As a consequence, the Editorial Board has lost confidence in the reliability of the reported findings and has decided to retract this publication.

This retraction was approved by the Editor-in-Chief of the journal *Cancers*.

The authors have been informed of this retraction but did not provide a comment on this decision.

## References

[1] Wang M., Wei K., Qian B., Feiler S., Lemekhova A., Büchler M.W., Hoffmann K. (2020). RETRACTED: HSP70–eIF4G Interaction Promotes Protein Synthesis and Cell Proliferation in Hepatocellular Carcinoma. Cancers.

[2] Wang M., Wei K., Qian B., Feiler S., Lemekhova A., Büchler M.W., Hoffmann K. (2020). Correction: Wang, M., et al. HSP70–eIF4G Interaction Promotes Protein Synthesis and Cell Proliferation in Hepatocellular Carcinoma. *Cancers* **2020**, *12*, 2262. Cancers.

